# Electron-Impact Total Ionization Cross Sections of Molecular Ions

**DOI:** 10.6028/jres.105.032

**Published:** 2000-04-01

**Authors:** Yong-Ki Kim, Karl K. Irikura, M. A. Ali

**Affiliations:** National Institute of Standards and Technology, Gaithersburg, MD 20899-0001; Department of Chemistry, Howard University, Washington, DC 20059

**Keywords:** electron-impact ionization, molecular ion, total ionization cross sections

## Abstract

The Binary-Encounter-Bethe (BEB) model for electron-impact total ionization cross sections of neutral molecules has been modified for molecular positive ions. The total ionization cross sections for $H_{2}^{+}$, $N_{2}^{+}$, CD^+^, and CO^+^ from the modified BEB formula are compared to available experimental data. The theory is in good agreement with experimental data on $H_{2}^{+}$, $N_{2}^{+}$, and CD^+^, but the experimental data by Belic et al. on $N_{2}^{+}$, and CO^+^ are lower than the theory. The large difference between the theory and experiment on CO^+^ is a strong indication of the dominance of the dissociative ionization channel, CO^+^→C^+^+O^+^, which was not included in the experiment.

## 1. Introduction

Ionization cross sections for atomic and molecular ions are among the essential data needed in the study of plasmas in fusion devices as well as in a diverse class of mass spectrometric studies of molecules. The Binary-Encounter-Bethe (BEB) model [1] has been successful in generating reliable total ionization cross sections of small and large molecules [2–6]. The BEB model combines a modified form of the Mott cross section with the asymptotic form of the Bethe theory (i.e., high incident energy *T*) for electron-impact ionization of a neutral atom or molecule.

In this article, we report a slight modification of the original BEB formula in order to adapt it to molecular ions. Almost all stable molecular ions are singly charged. The present modification has been found to reproduce known total ionization cross sections of singly charged ions, both atomic and molecular. We summarize the theory in Sec. 2, compare the theoretical cross sections to available experimental data in Sec. 3, and present our conclusion in Sec. 4.

## 2. Summary of Theory

The BEB formula for ionizing an electron from a molecular orbital of a neutral molecule by electron impact has a simple form [1]:

$$\sigma_{\mathrm{BEB}}=\frac{S}{t+u+1}\left[\frac{\ln t}{2}\left(1-\frac{1}{t^{2}}\right)+1-\frac{1}{t}-\frac{\ln t}{t+1}\right],$$ (1)

where *t* = *T*/*B*, *u* = *U*/*B*, *S* = $4\pi a_{0}^{2}$ *N R*^2^/*B*^2^, *a*_0_ is the Bohr radius (= 0.5292 Å), *R* is the rydberg energy (= 13.6057 eV), *T* is the incident electron energy, and *N*, *B*, and *U* are the electron occupation number, the binding energy, and the average kinetic energy of the orbital, respectively.

In Eq. (1), the terms in the square brackets have sound theoretical origin based on the Mott theory and the Bethe theory. However, the denominator *t*+*u*+1 is based on a plausible, but less rigorous argument, i.e., the effective kinetic energy of the incident electron seen by the bound target electron should be the incident electron energy *T* plus the potential energy *U*+*B* of the target electron [7]. Hence the *T* in the denominator of the original Mott and Bethe theories was replaced by *T*+*U*+*B*, or *t*+*u*+1 in Eq. (1), where *B* is used as the energy unit. This modification can be considered qualitatively as a simple scheme to introduce *T*-dependent electron correlation between the incident and target electrons.

The net effect of using *t*+*u*+1 instead of *t* in the denominator of Eq. (1) is to reduce substantially the cross section near the ionization threshold. This modification was found not only to be effective but also absolutely necessary to bring the theory in agreement with reliable experimental ionization cross sections near the threshold for many neutral atoms and molecules.

For singly charged ions, however, we noticed that this choice of the denominator reduced ionization cross sections too much near the threshold. This is qualitatively understandable because the Bethe theory and the Rutherford cross section, which is the Mott cross section without the electron exchange terms, increase when the target is an ion. These cross sections increase as *q*^2^, where *q* is the net charge of the target [8]. There are, however, no quantitative guidelines to modify the BEB model for ionized targets. We found that when we replaced *u*+1 by (*u*+1)/2 in Eq. (1), the slightly modified BEB formula reproduced known ionization cross sections for singly charged atomic and molecular ions, i.e.,

$$\sigma_{\mathrm{ion}}=\frac{S}{t+\left(u+1\right)/2}\left[\frac{\ln t}{2}\left(1-\frac{1}{t^{2}}\right)+1-\frac{1}{t}-\frac{\ln t}{t+1}\right].$$ (2)

Equation (2) for singly charged ions is as simple as the BEB formula for neutral targets, Eq. (1), and does not require any more input data than the original BEB formula.

In addition, we also have a more detailed version of the BEB formula when a quantity *Q* related to the continuum oscillator strength, d*f*/d*E* with photon energy *E* is known for each orbital [1]:

$$Q=\frac{2BM_{\mathrm{ion}}^{2}}{NR}$$ (3)

where

$$M_{\mathrm{ion}}^{2}=\int_{B}^{\infty }\frac{R}{E}\frac{df}{dE}dE.$$ (4)

When *Q* is known, then Eq. (2) is replaced by

$$\sigma_{Q_{\mathrm{ion}}}=\frac{S}{t+(u+1)/2}\left\{\frac{Q\ln t}{2}\left(1-\frac{1}{t^{2}}\right)+(2-Q)\left[1-\frac{1}{t}-\frac{\ln t}{t+1}\right]\right\}.$$ (5)

The value of *Q* is known for single orbital targets such as H, He, H-like ions, $H_{2}^{+}$, and H_2_ from either theory or photoionization experiments. We shall refer to this form of the cross section as the BEQ formula. The BEB formula, Eq. (1), was obtained by assuming *Q* = 1 when d*f*/d*E* for each orbital was not available.

## 3. Results and Discussion

**Fig. 1:**
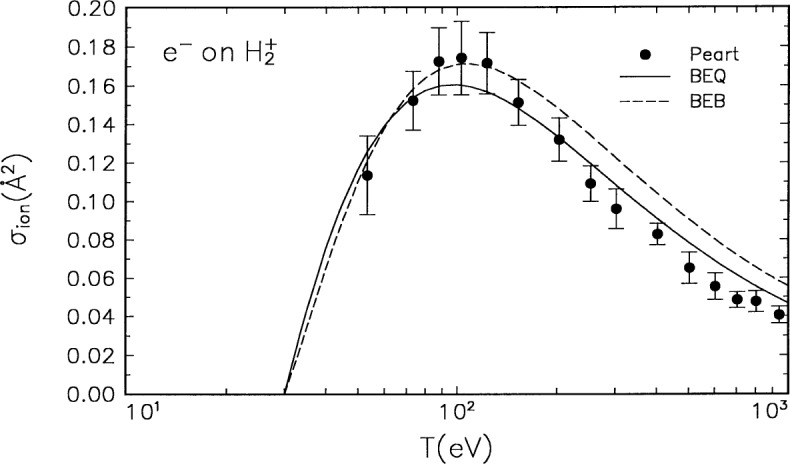
Comparison of the BEB and BEQ cross sections to experiment for $H_{2}^{+}$. Solid curve, BEQ cross section; dashed curve, BEB cross section; circles, experimental data by Peart and Dolder [9]. The ordinate is the ionization cross section, and the abscissa is the incident energy. The experimental uncertainties quoted by Peart and Dolder include uncertainties from both systematic and random effects.

**Fig. 2:**
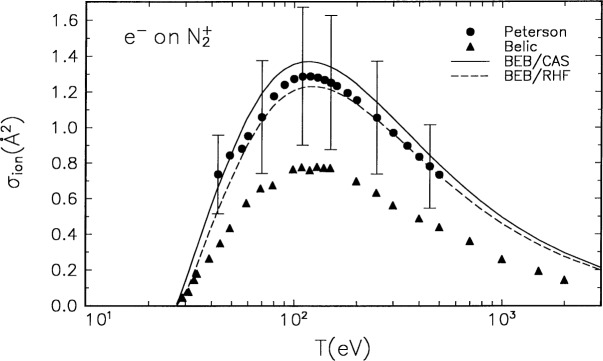
Comparison of the BEB cross section to experiments for $N_{2}^{+}$. Solid curve, BEB cross section using the constants from the restricted open-shell Hartree-Fock wave function; dashed curve, BEB cross section using the constants from the complete-active-space SCF wave functions; circles, experimental data by Peterson et al. [10]; and triangles, data by Belic et al. [12] and Siari et al. [11]. The two sets of experimental data represent slightly different physical quantities (see text). See Fig. 1 caption for other legend.

**Fig. 3:**
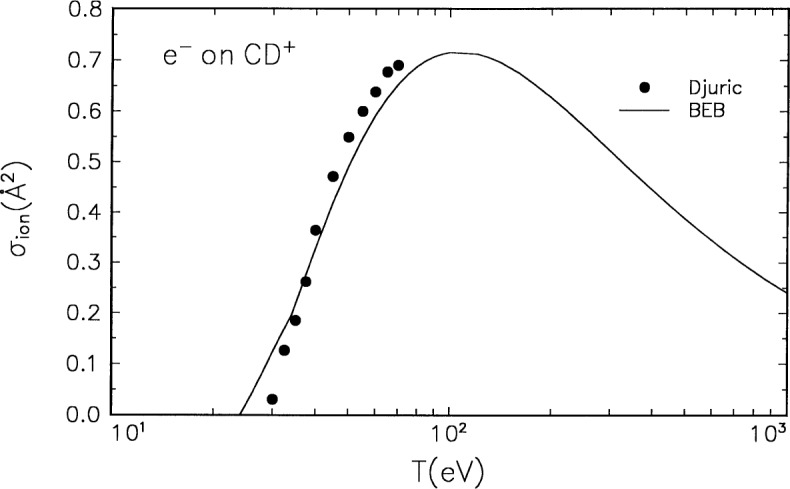
Comparison of BEB cross section to experiment for CD^+^. Solid curve, BEB cross section for CH^+^; circles, experimental data by Djurić et al. [13] for CD^+^. See Fig. 1 caption for other legend.

**Fig. 4:**
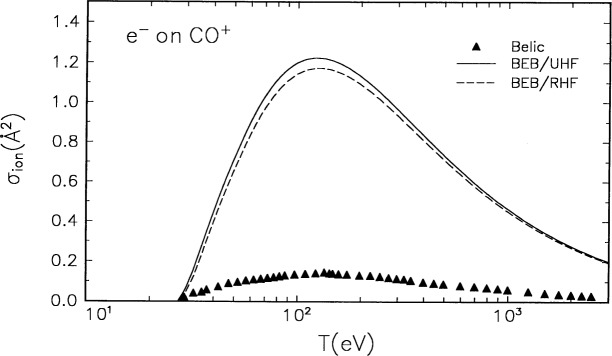
Comparison of BEB cross section to experiment for CO^+^. Solid curve, BEB cross section using a restricted open-shell Hartree-Fock wave function; dashed curve, BEB cross section using an unrestricted open-shell Hartree-Fock wave function; triangles, experimental data by Belic et al. [14]. See Fig. 1 caption for other legend.

We compare the BEB cross sections for $H_{2}^{+}$, $N_{2}^{+}$, CD^+^, and CO^+^ based on Eq. (2) to available experimental data [9–14] in Figs. 1–4. Most molecular constants *B* and *U* were calculated using the 6–311+G(d,p) basis set in the GAMESS code [15]. Since we were aware that electron correlation could increase the theoretical cross sections primarily through smaller *B* values [5], we obtained an alternative set of *B* and *U* for $N_{2}^{+}$ orbitals with low binding energies from complete-active-space (CAS) wave functions using the MOLPRO code [16]. Also, our experience with the BEB model indicated that it was important to use an accurate value of the lowest ionization energy (IE) to obtain reliable cross sections near the threshold. For this purpose, we found the IE from the frozen-core coupled cluster theory to be sufficient [17–18] (single- and double-excitation operators [19] with a perturbative estimate of the contribution from the triple-excitation operator [20], using Dunning’s correlation-consistent valence-triple-zeta basis set [21]). Theoretical results and comparisons to experimental data presented in this article are also available on a NIST web page for electron-impact ionization cross sections at *http://physics.nist.gov/ionxsec.*

In general, when an electron collides with a molecular ion we get

$$e^{-}+{\mathrm{AB}}^{+}\to {A+B}^{+}+e^{-}\;\mathrm{or}\;A^{+}+{B+e}^{-},$$ (a)

$$e^{-}+{\mathrm{AB}}^{+}\to {\mathrm{AB}}^{++}+2e^{-};\;A^{+}+B^{+}+2e^{-};\;A^{++}+{B+2e}^{-};\;\mathrm{or}\;{A+B}^{++}+2e^{-}.$$ (b)

Processes (a) are dissociation without ionization, while processes (b) are the ionizing events described by the BEB model. The model calculates the sum of all processes in (b) that lead to the ejection of a bound electron. The model can also account for double or higher multiple ionization resulting from the Auger decay of inner-shell holes [6], although the model is unable to account for the simultaneous ejection of two or more electrons from a single molecular orbital. On the experimental side, it is difficult to distinguish processes (a) from processes (b) in a single experiment unless a coincidence measurement of all products is performed. The usual experimental procedure is to measure the cross section for producing any ion, i.e., (a) + (b). Then, processes (a) are measured separately, and subtracted from the total ion production cross section. This subtraction introduces large uncertainties in the resulting experimental ionization cross sections.

### **3.1** $H_{2}^{+}$

The molecular orbital constants *B*, *U*, and *N* for $H_{2}^{+}$ are listed in Table 1, and the BEB and BEQ cross sections are compared in Fig. 1 to the experimental data by Peart and Dolder [9]. The value of *Q* can be derived by subtracting the equivalent of $M_{\mathrm{ion}}^{2}$ for discrete excitations [22], which is known as $M_{exc}^{2}$ and is equal to 0.7081, from the equivalent of $M_{\mathrm{ion}}^{2}$ for total inelastic collisions [23], which is commonly known as $M_{tot}^{2}$ and is equal to 0.8277. The resulting value of $M_{\mathrm{ion}}^{2}$ = $M_{tot}^{2}$ – $M_{exc}^{2}$ = 0.1196 leads to *Q* = 0.5275 for $H_{2}^{+}$. The comparison in Fig. 1 shows that the BEQ cross section is in slightly better agreement with experiment, as the BEQ formula contains more accurate information about the ionization continuum than the BEB formula.

### **3.2** $N_{2}^{+}$

The molecular orbital constants *B*, *U*, and *N* for $N_{2}^{+}$ are listed in Table 1, and the BEB model is compared in Fig. 2 to the experimental data by Peterson et. al [10] and by Siari et al. [11] and Belic et al. [12]. The experimental data by Peterson et al. are the difference between the total cross section for the production of N^+^ measured by Van Zyl and Dunn [24] and the dissociative excitation

$$N_{2}^{+}+e^{-}\to N^{+}+{N+e}^{-}$$ (6)

measured by Peterson et al. Although this difference, which is shown in Fig. 2, is in excellent agreement with the BEB cross section, the experimental difference counts the symmetric dissociation

$$N_{2}^{+}+e^{-}\to N^{+}+N^{+}{+2e}^{-}$$ (7)

twice because this process produces two N^+^ ions. However, the experimental data by Belic et al. [12] in Fig. 2 counts the symmetric ionization process [Eq. (7)] only once. The peak value of the cross section for the process [Eq. (7)] measured by Belic et al. [12] is about 0.25 Å^2^. If we reduce the data by Peterson et al. [10] by this amount, the lower half of their error bars will overlap with the data by Belic et al., which are estimated to have (5 to 6) % combined relative uncertainties due to systematic and random effects [11,12].

The BEB model counts each ionizing event only once. However, the theory assumes that every energy transfer exceeding the lowest IE will result in ionization, as all binary-encounter type theories do. For molecules, this assumption is a poor one because molecules can dissociate without ejecting an electron as in the dissociative excitation process [Eq. (6)] even when it receives an energy transfer exceeding the lowest IE. In short, the BEB model is likely to overestimate the total ionization cross section. Our experience with CF_4_ [5] indicates that the BEB cross section derived from correlated molecular wave functions is likely to be (5 to 10) % higher at the peak than the correct peak value.

### 3.3 CD^+^

The molecular orbital constants for CD^+^ are listed in Table 1, and the BEB cross section is compared in Fig. 3 to the experimental data by Djurić et al. [13]. The experimental ionization cross section was deduced by subtracting the cross section for dissociative excitation measured by Forck [25] from the total D^+^ production cross section measured by Djurić et al. [13]. The BEB cross section is insensitive to isotopes, and the theoretical cross section is for CH^+^, while the experiment was done with deuterium. No uncertainty estimates are available for the experimental data.

### 3.4 CO^+^

The molecular orbital constants for CO^+^ are listed in Table 1, and the BEB cross section is compared in Fig. 4 to the experimental data by Belic et al. [14]. The molecular constants for the BEB cross section were determined by using two different molecular wave functions for the molecular ion. Both sets of constants produce basically the same cross section. The experiment by Belic et al. [14] left out an important ionization channel:

$${\mathrm{CO}}^{+}+e^{-}\to C^{+}+O^{+}+2e^{-}.$$ (8)

Their experiment measured the cross section for the production of doubly charged ions, CO^++^, C^++^, and O^++^. As is shown in Fig. 4, the experimental cross section by Belic et al. [14], who estimate the combined relative uncertainties from systematic and random effects to be (5 to 8) %, is almost an order of magnitude lower than the present theory. The neutral molecules CO and N_2_ are isoelectronic, and their ionization cross sections are similar in magnitude and shape [2]. Hence, we expect ionization cross sections of CO^+^ and $N_{2}^{+}$ to be also similar in shape and magnitude. Such similarity is observed in the BEB cross sections in Figs. 2 and 4. The discrepancy between the theory and experiment in Fig. 4 indicates that the missing ionization process [Eq. (8)], which is equivalent to the symmetric dissociative ionization process [Eq. (7)] in a homopolar diatomic molecular ion, is the dominant mode for ionizing CO^+^.

## 4. Conclusion

We have shown that a simple modification, Eq. (2), of the BEB formula for neutral molecules, Eq. (1), produces reliable electron-impact total ionization cross sections for $H_{2}^{+}$ and CD^+^. On the other hand, the experimental data on $N_{2}^{+}$ by Belic et al. [12] are lower than the modified BEB cross section, while the experimental data deduced from the experiments by Van Zyl and Dunn [24] and by Peterson et al. [10] are in good agreement with the present theory taking into consideration the fact that the data by Peterson et al. counted the symmetric ionization process [Eq. (7)] twice, whereas the BEB cross section with the CAS wave function is likely to be (5 to 10) % too high as explained in Sec. 3.2.

Belic et al. [14] measured only part of the ionization cross section for CO^+^ that produces doubly charged ions. Judging from the large difference (see Fig. 4) between the present theory and the data by Belic et al. [14], it is likely that the major part of the ionization cross section of CO^+^ is in the “symmetric” dissociative ionization process [Eq. (8)], unlike the case for $N_{2}^{+}$, whose cross section for nonsymmetric ionization is about twice higher than that for the symmetric ionization [12].

## Figures and Tables

**Table 1:** Molecular orbitals, electron binding energy *B*, kinetic energy *U*, electron occupation number *N*, and the dipole quantity *Q* for $H_{2}^{+}$, $N_{2}^{+}$, CD^+^, and CO^+^. Wave functions: RHF stands for restricted Hartree-Fock, CAS means complete active space, and UHF is unrestricted Hartee-Fock.

Molecule	MO	*B*(eV)	*U*(eV)	*N*	*Q*
$H_{2}^{+}$, RHF	1σ	30.005^a^	16.3999	1	0.5275
$N_{2}^{+}$, RHF	1σ_g_	440.346	602.261	2	1
	1σ_u_	440.272	603.049	2	1
	2σ_g_	51.209	69.761	2	1
	2σ_u_	31.475	68.262	2	1
	1π	27.180^b^	48.672	4	1
	3σ_g_	30.577	62.221	1	1
$N_{2}^{+}$, CAS	1σ_g_	440.346	602.261	2	1
	1σ_u_	440.272	603.049	2	1
	2σ_g_	48.049	69.761	2	1
	2σ_u_	27.571	68.262	2	1
	1π	27.18^b^	48.672	4	1
	3σ_g_	26.175	62.221	1	1
CD^+^, RHF	1sσ	322.169	437.171	2	1
	2sσ	34.131	40.531	2	1
	2pσ	24.07^b^	38.199	2	1
CO^+^, RHF	1σ	573.418	794.761	2	1
	2σ	321.206	436.345	2	1
	3σ	52.344	81.881	2	1
	4σ	30.975	78.436	2	1
	1π	27.663	55.946	4	1
	5σ	27.3^a^	48.733	1	1
CO^+^, UHF	1σ	573.729	794.715	2	1
	2σ	321.916	436.120	2	1
	3σ	52.857	81.470	2	1
	4σ	32.263	73.082	2	1
	1π	28.311	56.083	4	1
	5σ	27.3^a^	60.505	1	1

a Ionization energies deduced from experimental data.

b Ionization energies from the coupled-cluster method [17–21].
